# Care at the first postnatal hour in two hospitals of the Adequate Birth Project: qualitative analysis of experiences in two stages of the Healthy Birth research

**DOI:** 10.1186/s12978-022-01540-5

**Published:** 2023-01-12

**Authors:** Maysa Luduvice Gomes, Lucia Regina de Azevedo Nicida, Débora Cecília Chaves de Oliveira, Andreza Rodrigues, Jacqueline Alves Torres, Amanda da Trindade Dias Coutinho, Beatriz da Silva Soares de Souza Cravo, Juliana Guimarães Dantas, Thays Basílio Oliveira, Patrick Brandão, Rosa Maria Soares Madeira Domingues

**Affiliations:** 1 Anna Nery School of Nursing, Federal University of Rio de Janeiro (UFRJ), Rio de Janeiro, RJ Brazil; 2 Faculty of Nursing, Rio de Janeiro State University (UERJ), Rio de Janeiro, RJ Brazil; 3 Collective Health from National Institute of Women, Children and Adolescents Health Fernandes Figueira (IFF), Oswaldo Cruz Foundation (Fiocruz), Rua Fabio Luz, 275. Bl. 5, Aptº 707, Rio de Janeiro, RJ 20725-232 Brazil; 4 Public Health at Sérgio Arouca National School of Public Health, Oswaldo Cruz Foundation (Fiocruz), Rio de Janeiro, RJ Brazil; 5 Institute for Healthcare Improvement, Rio de Janeiro, RJ Brazil; 6 Laboratory of Clinical Research in STD/AIDS, Evandro Chagas National Institute of Infectology, Oswaldo Cruz Foundation (Fiocruz), Rio de Janeiro, RJ Brazil; 7 Collective Health from National Institute of Women, Children and Adolescents Health Fernandes Figueira (IFF), Oswaldo Cruz Foundation (Fiocruz), Rio de Janeiro, Brazil

**Keywords:** Women, Birth, Health evaluation, Hospitals, Private

## Abstract

**Background:**

The Adequate Childbirth Project (PPA) is a quality improvement project that aims to enhance normal delivery and reduce cesarean sections with no clinical indication in the Brazilian supplementary health care system. This study aims to analyze the care model of the first postpartum hour in hospitals that participated in the PPA.

**Methods:**

Qualitative analysis based on the narrative of 102 women attended at two hospitals participating in the evaluative “Healthy Birth” research that analyzed the degree of implementation and the effects of the PPA. We assessed three practices within the first hour after delivery: skin-to-skin contact, breastfeeding and appropriate clamping of the umbilical cord. Data was collected through semi-structured interviews by telephone and submitted to thematic content analysis.

**Results:**

The categories that emerged from the analysis of the results were “Dimension of time and care expressed in the lived experience” and “Interferences in care in the first hour of life”. In the first category, women reported that in the first hour after delivery the newborn was placed on the mother's chest, but the length of time of the newborn's stay in skin-to-skin contact was less than one hour. This experience, even in a shorter period of time, was said to be positive by the women interviewed. Two barriers were observed: interruption of skin-to-skin contact for neonatal care and the transfer to the recovery room, both separating baby from mother without observing the duration of the "golden hour". It was identified that a process of improvement of the quality of care for childbirth is underway, with a gradual incorporation of recommended practices for care in newborn's first hour of life.

**Conclusions:**

Women reported access to the three care practices at two hospitals participating in the PPA quality improvement project. All practices were valued by women as a positive experience and should be promoted. Information during antenatal care to increase women´s autonomy, review of hospital practices to reduce barriers, and support from health care providers during the first hour after birth are needed to improve the implementation of these practices and access to their health benefits.

## Plain language summary

This study aims to analyze the care model of the first postpartum hour offered by two hospitals participating in the Adequate Childbirth Project (PPA), a quality improvement project to enhance normal delivery and reduce unnecessary cesarean sections in Brazilian private hospital. It is a qualitative analysis, based on the narrative of 102 women attended at two hospitals participating in the PPA. Categories that emerged from the analysis: “First hour; dimension of time and care expressed in the lived experience” and “Interferences in care in the first hour of life”. Most women expressed a chronological time of skin-to-skin contact far from the ideal recommended in the first postpartum hour; however, they valued the experience and its meaning. Two barriers were observed in this care process: the interruption of skin-to-skin contact for neonatal care and the transfer to the recovery room, without observing the duration of the "golden hour". We can conclude that women evaluated the service positively, with indications that point to the sustainability of the PPA. Information during antenatal care to increase women´s autonomy are needed to improve the implementation of these practices and access to their health benefits.

## Background

Studies on childbirth and immediate postpartum care practices aiming at maternal and newborn care have shown that the application of a set of simple procedures in the first hour after birth provides significant benefits to mother’s and baby’s health, both immediately and in a long-term. An important reference in this theme is the document entitled: “Beyond survival: integrated delivery care practices for long-term maternal and infant nutrition, health and development” [1] first published in 2007 by the Pan American Health Organization / World Health Organization (PAHO / WHO), whose second edition, published in 2013, was translated and distributed by the Brazilian Ministry of Health [2] in the same year. This document highlights three practices to be applied together: (1) late clamping of the umbilical cord; (2) immediate skin-to-skin contact (SSC) between mother and newborn; (3) early exclusive breastfeeding initiation [1].

The appropriate time for clamping the umbilical cord is after the umbilical circulation ceases and the cord is flattened and without a pulse. Short-term benefits pointed out for late clamping are the increase of placental blood volume transfused to the baby (which would be associated with increased hematocrit, hemoglobin, blood pressure, cerebral oxygenation, and flow of red blood cells), lower incidence of intraventricular hemorrhage, and late sepsis (after the first week of birth) [1, 3, 4]. For mothers with a lesser amount of blood in the placenta, there is the short-term benefit of shortening the third period and decreasing placental retention [1]. Regarding long-term effects, the main benefits for preterm and / or low birth weight of newborns is an increase in hemoglobin at 10 weeks of age and for those born at term there is an improvement in hematological status (hemoglobin and hematocrit) from two to four months and an increase in iron stores up to 6 months of age [1, 5].

Active and reactive babies should be placed face down on the mother's abdomen or chest, maintaining direct SSC, immediately after birth for at least one hour. SSC regulates and maintains the baby's body temperature, improves cardiorespiratory stability and the effectiveness of the first breastfeeding, and contributes to increased breastfeeding rates in the first four months of life and longer duration of breastfeeding periods. SSC is also associated with the reduction of crying episodes and signs of stress in the newborn, in addition to strengthening the emotional bond between mother and baby [1, 5, 6].

Breastfeeding in the first hour after birth is one of the Ten Steps to successful breastfeeding established in the Baby-Friendly Hospital Initiative [7] and is intrinsically linked to SSC. Exclusive breastfeeding right after delivery has immediate benefits for the baby and the mother, such as the prevention of neonatal morbidity and mortality and the release of oxytocin, which causes uterine contraction contributing to the reduction of maternal bleeding and postpartum hemorrhage, which represents 25% of worldwide maternal mortality [1, 4].

Considering all the previously mentioned benefits, routine baby care procedures that may hamper the implementation of these best practices during the first hour after the delivery should be reviewed. In 2015, the Adequate Childbirth Project (“Projeto Parto Adequado”—PPA)—a quality improvement project that aims to improve vaginal delivery, reduce cesarean sections without clinical indication, and improve care for newborns—was implemented in the Brazilian supplementary health system [8, 9]. This study aims to analyze the model of care in the first hour of the postpartum period in two hospitals participating in the PPA.

## Methods

The Healthy Birth study is an evaluative research, conducted by the Sérgio Arouca National School of Public Health of the Oswaldo Cruz Foundation, which aims to assess the degree of implementation and the effects of the PPA. The PPA was conducted in three phases, with Phase 1 being developed between May 2015 and November 2016, when the proposed intervention was tested in 35 public and private hospitals.

The Healthy Birth study used a mixed methods approach, with a cross-sectional design in the quantitative component. Quantitative data were collected in two moments: the first, Moment 1 (M1), from March 2017 to August 2017, included twelve private hospitals that participated in the first phase of the PPA, aimed at evaluating the degree of implementation of the PPA. Inclusion criteria were: hospital location (according to regions of the country); type of hospital (hospitals owned or not owned by health plan operators); and hospital performance (hospital performance was classified as “good” or “bad”, as assessed by the PPA coordination). In each maternity hospital, a sample size of approximately 400 women was calculated, aiming to detect a 10% reduction in the proportion of cesarean sections. All puerperal women with a hospital delivery of a live birth, of any gestational age or birth weight, or of a stillborn fetus, with gestational age ≥ 22 weeks or weight ≥ 500 g, were considered eligible for the study. Women with hearing impairment, foreigners who did not speak Portuguese, women with pregnancies with 3 or more fetuses, and women hospitalized for judicial termination of pregnancy were considered ineligible. In each hospital, women were invited to participate in the study consecutively until the planned sample was reached.

The second moment, Moment 2 (M2), aimed to assess the sustainability of the PPA implementation. Quantitative data collection took place from March 2018 to August 2018, with only eight of the twelve initial hospitals. Four hospitals were excluded due to geographic location and similar results. In each of these eight hospitals, the methodological procedures of the first moment were repeated, with interviews of approximately 400 women in each hospital and extraction of data from women's and newborn records.

**Fig. 1:**
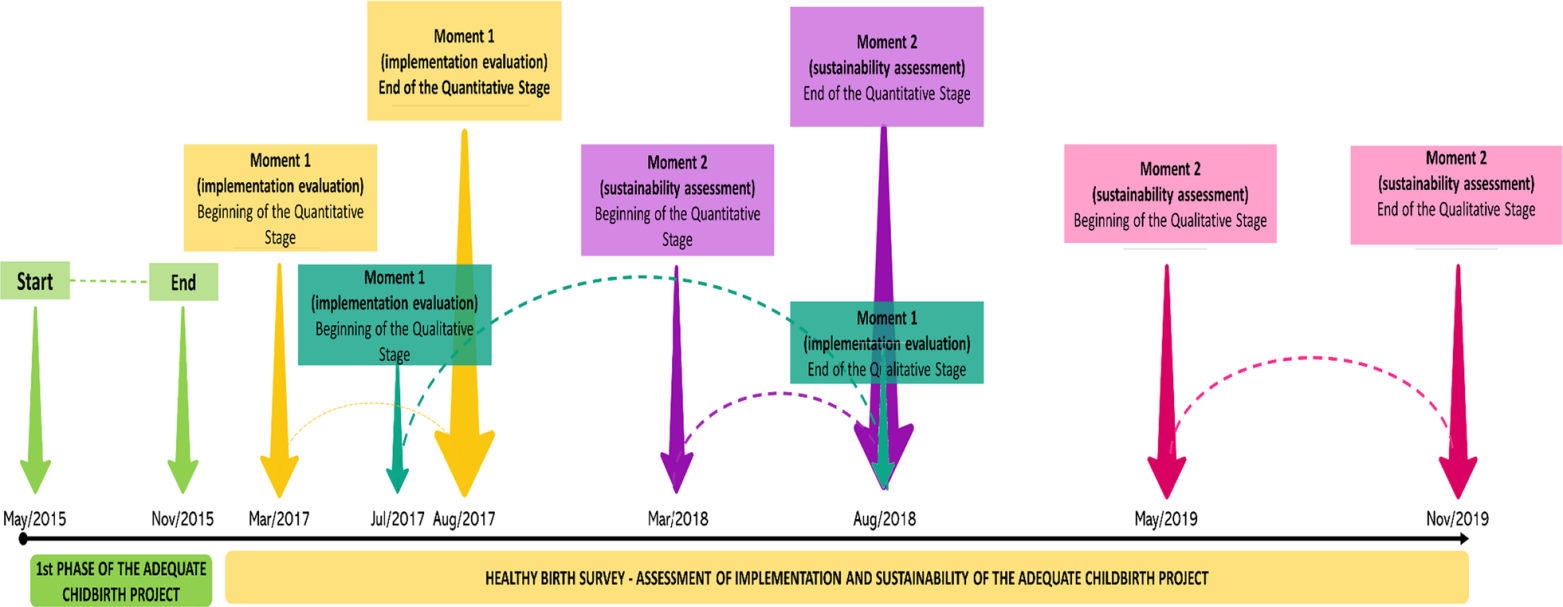
Timeline of the Adequate Childbirth Project and the Healthy Birth research. Source: Created by the authors

The qualitative component also took place in two moments. Data collection in M1 took place from July 2017 to August 2018, in eight out of the twelve hospitals that had the best results under the degree of implementation. Data collection in M2 took place from September 2018 to November 2019, in four of the eight hospitals in M2, with better implementation performance. In both M1 and M2, the qualitative component included hospital managers, health professionals directly involved in childbirth (doctors and nurses), non-participant observation, and finally, telephone contact with the women approximately six months after delivery for an in-depth interview (Fig. 1). More details about data collection, contextual aspects and protocols established by the Healthy Birth research can be found in Torres et al. [8] and Domingues et al. [10].

**Fig. 2:**
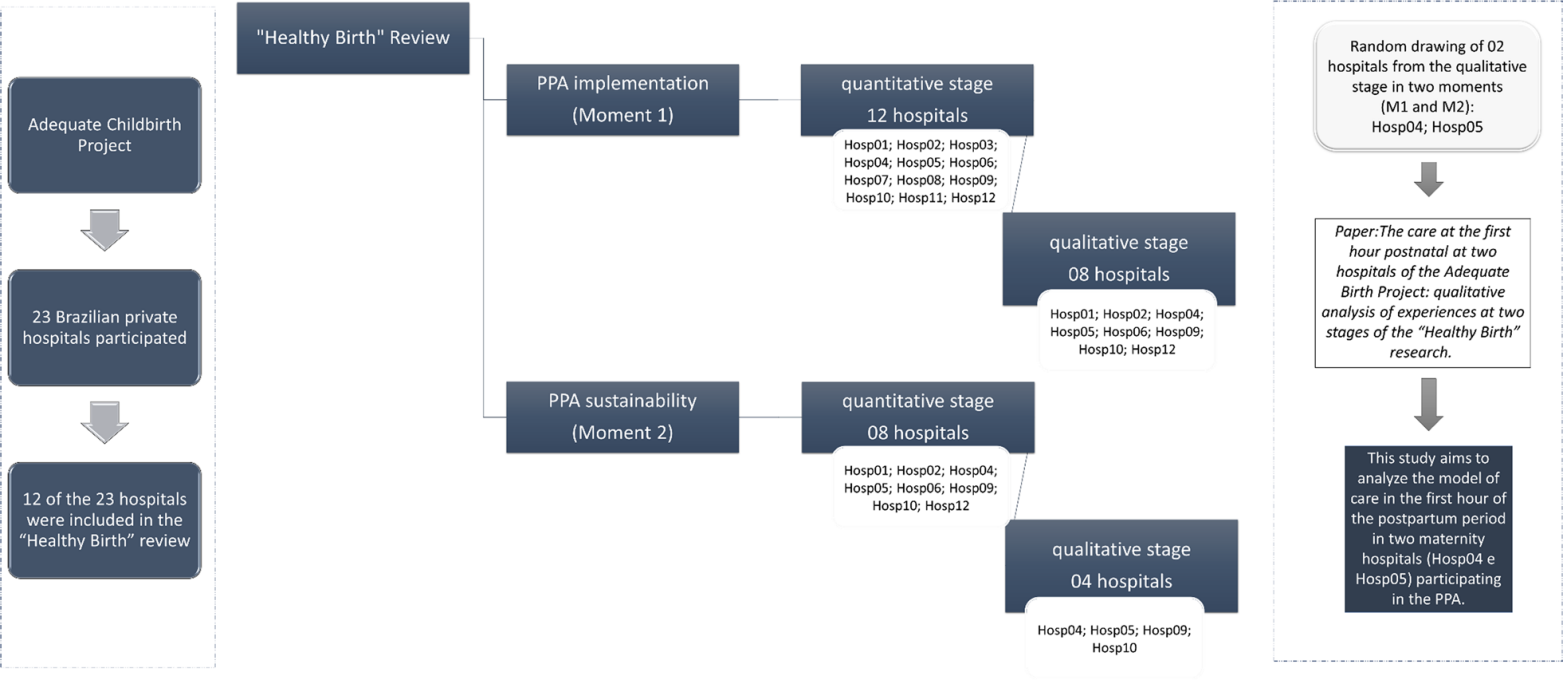
Selection process of hospitals participating in the study. Source: Created by the authors

In this analysis, we used data of the qualitative component, from two hospitals (Hosp 4 and Hosp 5) randomly chosen among those that participated in M1 and M2 (Fig. 2). Hospital 04 was founded in 2001, is a high complexity hospital, located in the Southeast region of Brazil and is not owned by a health plan operator. It is a research and teaching hospital with residencies in Anesthesiology and Intensive Care Medicine. The hospital's website contains a space for women's health providing information on different topics including preparation for childbirth and postpartum recovery, among others. The cesarean section rate ranged from 65.5% in 2017 to 60.9% in 2019. Hospital 5 was founded in 1971, is a high complexity hospital located in the South region of Brazil and is owned by a health care operator. The cesarean section rate ranged from 70.6% in 2017 to 72.3% in 2019.

**Fig. 3:**
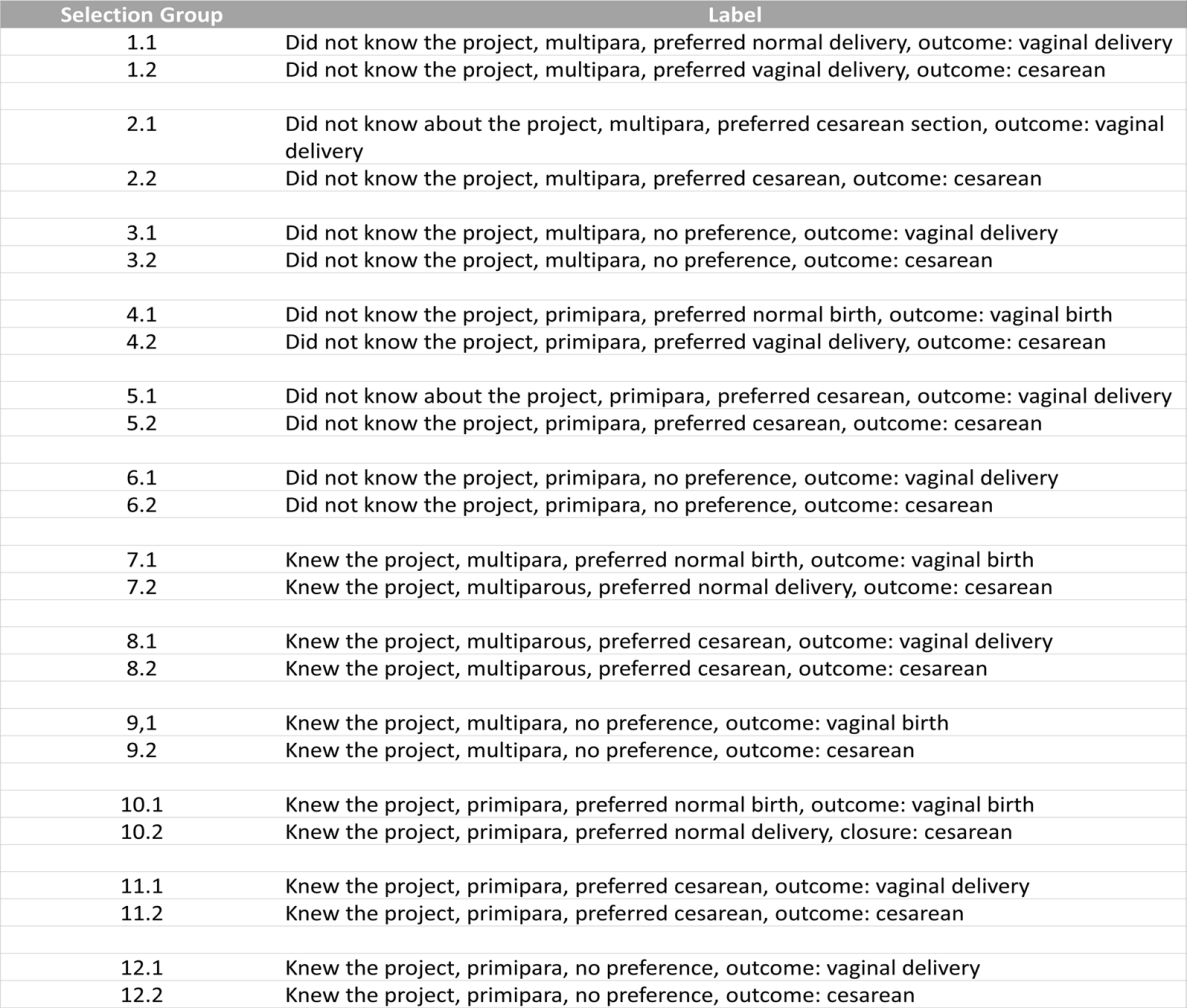
Selection process of women for the in-depth interview through telephone calls. Source: Created by the authors

In both hospitals, we only used data from telephonic interviews with women. For the selection of women, an initial list was drawn up based on data collected in the quantitative stage of the research including 804 women for M1 and 729 for M2. The women were classified into 24 groups considering the variables “knowledge about the project” (yes or no), “parity” (primipara or multipara), “preference for the type of delivery” (vaginal, cesarean section, no preference), and “type of delivery” (vaginal or cesarean section) (Fig. 3). Women from all groups were randomly selected reaching a final number of 102 interviewees (M1: Hosp 04—36 interviewees, Hosp 05—19 interviewees / M2: Hosp 04—23 interviewees, Hosp 05—24 interviewees).

### Data collection process

The in-depth interviews were conducted by telephone between six and twelve months after delivery. A semi-structured interview instrument was used and covered the axes of prenatal care, the selection and visit to the maternity ward, expectations and experiences about childbirth (in which aspects of postpartum care were included) and the PPA strategy. The interview guide was developed for this study and is published in Torres et al. [8].

The initial approach with the interviewees occurred by sending a message via the WhatsApp application, from which the invitation to conduct the interview was made. Upon acceptance of the invitation, telephone contact was made on a day and time chosen by the woman. The interviews were recorded with verbal authorization from women who had already signed a Free and Informed Consent Form (FICF) for all stages of the research at the time of their admission for labor and birth. Through this ethical consent form, which was read and signed by each woman, she was informed about the research objectives, the procedures that would be used, and about confidentiality. She was also informed about possible discomforts, risks and benefits, and that at any time she wished, the research could be interrupted. The FICF included the consent to be interviewed and audio recorded. Interview time varied around thirty minutes, according to each interviewee's desire to speak.

Seven interviewers received theoretical and practical training, which was conducted in a pilot study in one of the participating maternity hospitals, allowing the adjustment of the questionnaires and refinement of the procedures related to fieldwork. The interviews were transcribed by an independent professional and reviewed by the research team. In the following step, the interviews were imported into the MaxQda software, 2020.1 version, and their data were subjected to Thematic Content Analysis, according to Uwe Flick [11].

Composing the analysis process, the interviews were organized and encrypted according to the respective hospital, the woman's unique numbering and type of delivery. The cryptography used was identified only in the research dictionary in order to guarantee the anonymity of the interviewees.

**Fig. 4:**
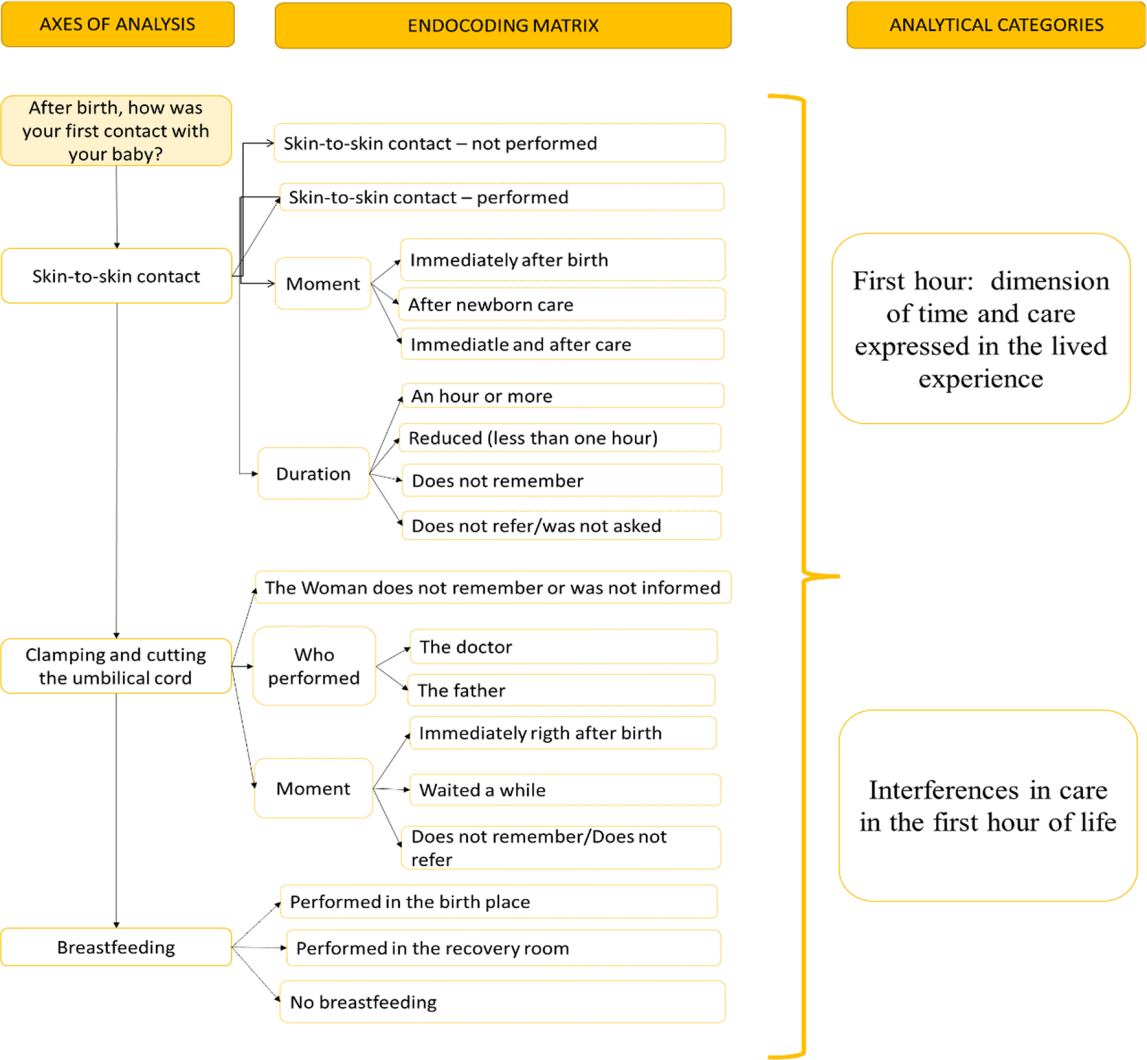
Summary matrix of the categories and the content analysis process. Source: Created by the authors

The code matrix was elaborated from the women's report on the care received in the first hour after birth in relation to skin-to-skin contact, clamping and cutting of the umbilical cord and breastfeeding, generating broad coding of segments. Subsequently, a synthesis of each coding was carried out in order to deepen the meaning of the explanations, a stage called axial coding. At this stage, the data were refined and then structured as a list of encodings by emerging classifications, based on the sense of the synthesized content. From this list of classified codes, inductive associations were carried out to create the final categories. This process includes the three techniques suggested by Flick, 2009 (explanation, synthesis and structure) for further categorization. The categories that emerged from this analysis process were: “Dimension of time and care expressed in the lived experience”, which reveals that the first postpartum hour (“golden hour”) is experienced by women due to the intensity of the moment and not in chronological time; and “Interferences in care in the first hour of life”, which reveals the barriers related to professional attitudes and hospital structures in the care in the first postpartum hour (Fig. 4).

For data interpretation, the interviewees' statements and their respective codifications were validated by members of the research group, in which there were also reflective co-participations on the interpretative procedures of the entire analytical phase [11]. We explored the factors of the care model proposed by the PPA for the first postpartum hour implemented by the hospitals and compared the improvement of care practices between the data collection periods M1 and M2.

## Results

### Participants profile

Table 1 shows the socio-demographic characteristics of women interviewed in the two phases of the qualitative component. Most women were between 20 and 34 years old, self-reported as white, had a high level of education (graduation and post-graduation), had paid jobs, were married or lived with a partner. The proportion of primiparous and multiparous women and of vaginal births and cesarean sections were similar, due to the sampling procedures adopted.

**Table 1:** Socio-demographic characteristics of participants per hospital and data collection period

	Implementation—M1 (n = 55)		Sustainability—M2 (n = 47)	
	Hospital 04 (n = 36)	Hospital 05 (n = 19)	Hospital 04 (n = 23)	Hospital 05 (n = 24)
	n (%)	n (%)	n (%)	n (%)
Age (years)				
Less than 20	–	–	–	1 (4.2)
20–34	25 (69.4)	16 (84.2)	13 (56.5)	19 (79.2)
35 +	11 (30.6)	3 (15.8)	10 (43.5)	4 (16.7)
Self-reported skin color				
Black	-	1 (5.3)	1 (4.3)	1 (4.2)
White	25 (69.4)	15 (78.9)	14 (60.9)	17 (70.8)
Brown/Yellow/Indigenous	11 (30.6)	3 (15.8)	8 (34.8)	6 (25)
Level of Education				
Elementary School	–	–	–	1 (4.2)
High School	3 (8.3)	7 (36.8)	4 (17.4)	7 (29.2)
Incomplete Graduation	3 (8.3)	1 (5.3)	1 (4.3)	2 (8.3)
Graduation	17 (47.2)	6 (31.6)	6 (26.1)	7 (29.2)
Post-graduation	13 (13.1)	5 (26.3)	12 (52.2)	7 (29.2)
Paid work				
Yes	30 (83.3)	18 (94.7)	22 (95.7)	17 (70.8)
Marital status				
Single	1 (2.8)	–	–	2 (8.3)
Married/Lives with partner	35 (97.2)	19 (100)	23 (100)	21 (87.5)
Separated/Divorced	–	–	–	1 (4.2)
Parity				
Primiparous	19 (52.8)	9 (47.4)	13 (56.5)	13 (54.2)
Multiparous	17 (47.2)	10 (52.6)	10 (43.5)	11 (45.8)
Type of delivery				
Cesarean section	17 (47.2)	8 (42.1)	10 (43.5)	11 (45.8)
Vaginal Birth (includes forceps and vacuum extractor)	19 (52.8)	11 (57.9)	13 (56.5)	13 (54.2)

### Axes of analysis

We analyzed the care in the first hour using the three axes of the interview: skin-to-skin contact, clamping and cutting of the cord, and breastfeeding.

### Skin-to-skin contact

The recommendation made by PAHO / WHO [1] is that skin-to-skin contact with a baby under normal conditions should be done immediately and continuously, for at least one hour from birth. Most participants in this study revealed that SSC was performed with the baby on their body without the use of clothing, but with variations as to the moment and duration.

"Yes. She came straight to my chest. (…). He gave her to me like this, naked, body to body””. (M1_Hosp05_VB)

“Wow! Exciting! (…) He was placed on my chest. (…) First we were just hugging him, then they passed a cloth while he was already next to me and was already with me, just hugged, on my chest. (…) His skin with my skin". (M2_Hosp04_VB).

Three types of SSC were identified:

Immediate SSC after birth

"The moment he was born, he came with umbilical cord and everything, he came to my chest (…)". (M1_Hosp05_VB)

"It was in the cesarean delivery room, I was immediately put in contact with him. (…) Skin on skin ". (M1_Hosp04_CS)

SSC performed only after newborn care

"The doctor took her from me, (…), showed her to me, the pediatrician took her to do the procedures, and then brought her to me. (…) on my chest, so she could feel my body and I could feel her (…). This contact was very nice". (M1_Hosp05_CS)

"First the doctor took the baby, showed him to me and then took him there to do (…) to weigh the baby, and then they brought me (…) [doing] that skin to skin (…). It was very nice ". (M1_Hosp05_CS)

Immediate SSC, interruption for care of the newborn and / or mother, then returns.

"He went straight to my chest. The whole time. He only left after some time. He left, I think for about five minutes, he was weighed and then he came back to my chest. Five minutes at the most, he stayed away." (M1_Hosp04_VB)

"(…) she put him on my chest first and then the pediatrician did a quick assessment, put him on the scale and gave him back to me. Then he stayed with me for a long time (…)". (M2_Hosp05_VB)

Regarding the duration of SSC practice, very few women mentioned having done it for an hour or more. Thus, the vast majority had a reduced SSC experience (less than one hour). In both M1 and M2, the interviewees' reports showed a lack of knowledge about SSC as a procedure that is part of the first hour of care, about how to perform it and the recommended duration and benefits.

"About four, five minutes ". (M1_Hosp04_CS)

"I think about twenty minutes. It was quite a long time". (M2_Hosp05_VB)

The time dedicated to the postpartum care of the mother, the care of the newborn and the transfer to the recovery room were factors that interfered in the duration of the SSC.

"(…) I stayed with her for about forty minutes, until she went for her exams". (M2_Hosp04_VB)

"It was quite a bit. While the doctor was doing the (…) he was closing the cesarean section, while they were doing the instrumentation count, all that time she stayed with me". (M2_Hosp05_CS)

Women who had a vaginal delivery reported immediate and continuous SSC more frequently than women who underwent a cesarean section. The skin-to-skin contact occurred mostly after the care of the newborn.

The immediate postpartum period is a time of transition and great intensity, and each woman and baby would need different timing to adapt to this moment. The women expressed in their narratives the duration of the first contact with doubts and imprecision, qualifying the time more by their impressions and meaning as reported below:

"It was wonderful, it was exciting, I remember it like it were today. She went straight onto my chest … Let me think here … I think a minute at most. Really fast. She came to my chest and she stopped crying on my chest, then they took her off my chest, she started crying again, to do all the procedures, you know, the measurements, to know if she was healthy and so on". (M1_Hosp04_CS)

"I remember it wasn't quick, but it didn't take long either. It took the time for us to feel, to caress, to take a picture and so on and then they took him away. About two minutes all of a sudden". (M2_Hosp05_VB)

A smaller number of participants reported that there was no skin-to-skin contact and most of these cases were associated with the existence of some complication. However, there were also reports in which there was no apparent reason for not performing the SSC.

"It was very fast, the pediatrician next to me warned me, so that I would be prepared, that in a short time they would show me the baby, I was already looking in expectation, waiting for the baby, the doctor showed him to me, I looked at his little face for a little while and soon after she passed him to the pediatrician to analyze and as he was born with a little bit of breathing difficulty, the pediatricians took him to the next room (…)". (M1_Hosp04_CS)

"He was naked, but he had a blue cloth underneath him". (M2_Hosp04_CS)

### Clamping and cutting the umbilical cord

We explored two dimensions related to the clamping and cutting of the umbilical cord: 1) the moment of clamping, followed by the cutting of the cord; and 2) the possible offer for the woman's partner or companion to perform the cut.

Fewer women commented on the umbilical cord practices when compared to SSC and breastfeeding. Among those who made comments about this practice, most reported that there was a waiting period before the clamping and cutting were performed.

"She waited a little bit, she put it on, she showed me, then she said if I wanted to keep her a little bit, she put her on me a little bit and she came back and cut the cord". (M2_Hosp05_CS)

"They waited for it to stop pulsing". (M2_Hosp04_VB)

Comparing the reports of women who had a vaginal delivery with those who had cesarean section, differences were found regarding the person who performed the cord cutting. When the birth occurred by cesarean section, the clamping and cutting of the cord was mostly performed by the doctor. Women did not demonstrate dissatisfaction with this practice if it was perceived as part of the necessary surgical procedures.

“She was my doctor, we even talked to him about it during prenatal care. She would have loved my husband to cut the cord, if I had a vaginal delivery. As it was a surgery, I would be open and so on, she thought it was risky. So, she was the one who cut the cord". (M1_Hosp04_CS)

“No, it was the doctor himself too. One of the things I wanted, one of the things I was hoping for, was that my husband cut the cord, to breastfeed right after he was born and so on, but the doctor wouldn’t let me do that, nothing. (The cord) was cut right away". (M1_Hosp04_CS)

There were very few women who knew that the cutting could be done by someone other than the doctor and were frustrated about how it happened.

"I wish my husband had cut the cord". (M2_Hosp04_CS)

“I had even read something about it that caught my attention, but he (the doctor) cut it after he was born". (M2_Hosp05_CS)

Women who had a vaginal delivery reported more often that clamping and cutting of the cord could be performed by the father, if this was their wish, and that these procedures were performed closer to the opportune moment. This result was found in both M1 and M2. Although there are no evidence-based recommendations for the cord cutting being performed by the woman's companion and few studies discussing its emotional value, the women's reports suggest it may be an important aspect for the participation and bonding of the newborn's father [12].

“My husband cut the cord. He cut it only after it stopped pulsing. It was in my birth plan, when she asked me". (M1_Hosp04_VB)

"He stayed after a long time the cord started to stop beating and then the doctor asked if my husband wanted to cut it. He said: I really want to, because I also cut the first one. Then he cut it". (M2_Hosp04_VB)

### Breastfeeding

In most reports, women did not identify breastfeeding as a practice that is part of the routine care and procedures in the first hour after birth. Some of them expressed lack of knowledge about how to do it and even a certain surprise at the possibility of breastfeeding at that moment.

“They taught me, I wasn't sure how to do it, then one of the nurses there taught me how to do it and the baby didn't know how to breastfeed and she was explaining me how to put the breast, how I had to open her mouth, she explained it". (M2_Hosp04_VB)

“I remember it was already placed on the spot at the birth. They took her out and put on my breast. She even started to rehearse a (…) it caught my attention that she already suckled. In the first hour, she already suckled. She seemed to already know how to suckle". (M2_Hosp05_CS)

As for the place where breastfeeding occurred, most women experienced the beginning of breastfeeding in the delivery room or in the operating room, some reported that it occurred in the recovery room, and few stated that the baby did not go to the breast in the first hour after birth. This profile was maintained in M1 and M2, in both vaginal delivery and cesarean section.

"They only cleaned, they didn’t bathe, and he already suckled (…) He suckled easily, it was quiet". (M1_Hosp04_VB)

“It was right there, in the small room. They showed me, put me in contact, then she suckled right there, during the surgery process, she suckled right there, she was very close, so I think it was immediate, like this". (M1_Hosp04_CS)

“So much so that as soon as she was born, she suckled an hour without stopping. They didn't wash her, nothing. They wrapped her in a cloth, put her on top of my chest, there they were still closing me ". (M2_Hosp05_CS)

In cases where breastfeeding was started soon after birth, it was sometimes interrupted to perform other procedures, either on the newborn or on the mother.

"Yes. I stayed with her, then the pediatrician came to do the procedures they do with the baby, then she went to measure and did the procedures with her and they gave her back to me, then I stimulated her to breastfeed ". (M1_Hosp05_VB)

“He must have stayed about five minutes, I know he did, then he got to breastfeed and then the pediatrician took him and went to weigh him." (M1_Hosp04_CS)

The transfer to the recovery room appears to be an obstacle for breastfeeding to occur during the first hour of care. However, in cesarean sections, the first hour after birth should coincide with the necessary time for surgical care.

"As far as I remember, I felt him well and he also breastfed, even afterwards. In the surgical center he already suckled. I think it was about half an hour, more or less, about forty minutes, because that was how long it took to finish the surgery". (M2_Hosp04_CS)

Some women stated that they were not able to breastfeed in the first hour and pointed out impediments, mainly related to the baby's adaptation to the extra-uterine environment.

“Yes, they tried but I had no milk. The milk only came afterwards". (M1_Hosp05_CS)

“He stayed on my chest, so I tried to breastfeed him, but he was too weak. He was born very weak, a very pronounced hypotonic, so I couldn't breastfeed him, but he stayed with me all the time". (M2_Hosp05_CS)

Women reported satisfaction and emotion with SSC and breastfeeding, no matter the time and the duration of these practices. Women measure time from the lived experience, a sense that was observed in vaginal deliveries as well as in cesarean sections.

"He waited for the cord to stop pulsating and then the father cut it, then he stayed with me for three minutes, around four minutes, after that he took her to be cleaned, he didn't bathe her, no. He just cleaned her up. Then, he gave her back to me, then he put her on my breast, she started to suckle properly, right there in the delivery room. It was really beautiful. I get emotional, because it was very nice. It was a blessing". (M1_Hosp04_CS)

The sharing of information appeared as an aspect that contributes to women’s autonomy so that they feel safe and confident to carry out the procedures in which they are protagonists.

“Yes. It was in the delivery room. It was right after I picked him up. The staff had already commented at the hospital course. Then, leaving the baby close to the breast, as soon as I put him to the breast, he already went with his mouth looking for me and suckled already”. (M2_Hosp05_VB)

"Yes [they talked about the PPA during the visit]. They talked about everything I had learned in the conversation circle, in the health plan and they talked about the importance of the golden hour, that my baby could stay with me after birth, that they didn’t bathe him in the first twenty-four hours of the baby's life, …everything I had heard in the conversation circle". (M2_Hosp04_CS)

As emerged in the reports concerning their experiences, lack of information and preparation for these practices restricted women's autonomy and freedom, putting them in a place of greater vulnerability in the relationship with the professionals and work processes and routines that occurred in their assistance. The women's speech reveals hindrances in the institutions as follows:

"(…) after I went to the recovery room, they brought [my son] to me and then, yes, he came to breastfeed, but I didn't have any orientation. I found that very complicated. There was no nurse by my side, no technician, no one to guide me, how would breastfeeding be, no one". (M1_Hosp05_CS)

"This is something that I realize today that could have been better. I don’t know if it was because he was at the limit of being premature, but he stayed very close to me for a short time, then the pediatrician took him right away to do the birth exams and so on”. (M2_Hosp04_CS)

## Discussion

Two categories of analysis emerged: “Dimension of time and care expressed in the lived experience” and “Interferences in care in the first hour of life”.

In the category “Dimension of time and care expressed in the lived experience”, many women had access to the three practices recommended by PAHO/WHO [1] and by the Brazilian Ministry of Health [2]. However, the “golden hour”, that is, the continuous contact of mother and baby immediately after birth, was not part of the routine care provided by the two analyzed hospitals, whether in M1 or M2.

Nonetheless, even with a shorter duration than recommended, women expressed the experience of this practice in an intense, pleasurable way, marked by emotion, giving it a dimension of time by the intensity of this lived experience, in the sense that sometimes a fleeting moment has a profound positive effect on the parenthood experience [13].

Putting the healthy baby in skin-to-skin contact and offering help so that the woman recognizes that her baby is ready to suckle her breast is the fourth step of the Baby-Friendly Hospital Initiative [7]. It is a conduct recommended to professionals because it is an important factor that interferes positively in the first hour of life [14–17]. Several studies in the obstetric area have deepened the understanding of these effects and their relationship with health benefits to mothers and babies, such as better adaptation of the baby to extra-uterine life, facilitating thermoregulation; adaptation of the newborn's blood glucose level; and promotion of breastfeeding and its duration, avoiding early weaning. This reaffirms the importance of skin-to-skin contact being carried out immediately after birth and lasting one hour [18–21]. If we consider that this information has been shared among medical and health professionals, with emphasis on those who work in childbirth care, this study identifies that it has not yet been routinely incorporated to the point that women perceive it as a practice that must be performed in its entirety, such as the duration of one hour uninterruptedly [18–22].

Women who had a cesarean section reported skin-to-skin contact, which is a positive finding, since previous studies have pointed out that SSC is not performed in this type of birth [23]. Considering the high rates of cesarean sections in Brazil, mainly in private hospitals, the performance of SSC in this type of birth shows that quality improvement projects, such as the PPA, can have positive effects on the adoption of best care practices.

Few women reported aspects related to the clamping and cutting of the cord, which suggests the need to provide information to women and families, as to enhance the knowledge of the benefits of late clamping and its positive effects on the health of the baby and its level of human development, which includes its association with a higher hemoglobin concentration and a lower incidence of childhood anemia [4, 24, 25].

Most women did not demonstrate knowledge of the beneficial effects of the evaluated practices but remembered the first contact with pleasure and satisfaction using terms such as “exciting”, “beautiful”, and “wonderful”. Neuroendocrine responses are activated during this first contact, with the release of oxytocin as a neuromodulator, contributing to the strengthening of the mother-baby bond, to lasting breastfeeding as a source of nutrition and survival of the newborn, among other benefits [22, 26]. Most likely due to the lack of knowledge of the benefits of these practices, no striking expressions of claim were observed, such as women demanding the use of these best practices in the first hour, as recommended by international and national guidelines. It also makes us wonder how professionals evaluate these procedures.

Facing and overcoming challenges related to women's health is a complex phenomenon that requires reflection and a joint effort between government, institutions, organizations, and the population, that in permanent struggle movements, like the one called as “humanization of childbirth”, strives to favor the dissemination of information, greater female empowerment and improvement in care conditions [27, 28].

In both hospitals, women interviewed in the second data collection moment (M2) were more concerned about these three practices and how they could have been performed. This finding suggests that both hospitals have probably worked to improve the implementation and maintenance of evidence-based practices recommended by the PPA. However, the lack of women's knowledge points to the need of more effective measures to allow their participation in the decision-making process regarding the care they will receive.

In the second category: “Interferences in care in the first hour of life”, many barriers were identified for SSC and breastfeeding in the first hour after birth, related to needs and complications of the baby and professional practices. The immediate care of the baby and the woman's transfer to the recovery room appeared as the main obstacles to SSC and breastfeeding in the first hour. In some cases, hospital practices with the baby had to coincide with the time of women care in the delivery or operation room. To do so, the pediatrician examines the baby and only afterwards, it comes to the mother. The surgical procedure per se appeared as an impediment to the cutting of the cord by the father. The change in these practices is complex, as they relate to many aspects of the childbirth care model in Brazil. For example, all births in these hospital services occur in the surgical or obstetric centers, which must be quickly released to allow its occupation by another parturient [2]. The transfer to the recovery room, one of the barriers identified to SSC and early breastfeeding, is a form to organize the patients flow in the hospital care service. This model of care is embedded in institutional values that define the setting in which the births are performed. Therefore, a vaginal delivery assisted in an operating room can be itself an obstacle to changing the model.

Changing the setting can cause changes in the practice model, as indicated in projects that encourage the adoption of PDP rooms, a model in which the pregnant woman is kept in the same room during pre-delivery, delivery and the puerperium [29–31]. This change of setting fosters changes in the hegemonic obstetric model, in which women are transferred to different pre-delivery, delivery and puerperium rooms, which interferes in the continuity of care as reported by some women.

## Conclusion

Women reported access to the three care practices within the first hour after delivery at two hospitals participating in the PPA quality improvement project. However, many barriers related to the organization of care were identified and indicate the need for further improvements. Skin to skin contact and breastfeeding in the first hour of life were affected by hospital and professional practices. Clamping and cutting of the cord was the least known practice by women and was perceived as part of the surgical procedure in case of cesarean section. All practices were valued by women as a positive experience and should be promoted. Information during antenatal care to increase women´s autonomy, review of hospital practices to reduce barriers, and support from health care providers during the first hour after birth are needed to improve the implementation of these practices and access to their health benefits.

## Data Availability

The datasets used during the current study are available from the corresponding author on reasonable request.

## Abbreviations

ANS: National Supplementary Health Agency
CS: Cesarean section
VB: Vaginal birth
PAHO: Pan American Health Organization
PPA: Adequate Childbirth Project (Projeto Parto Adequado)
SSC: Skin-to-skin contact
UNICEF: United Nations Children's Fund
WHO: World Health Organization
